# Agr2 in cancer and beyond: unraveling its role during protein synthesis, ER stress, and as a predictive biomarker

**DOI:** 10.1007/s11010-025-05318-8

**Published:** 2025-06-05

**Authors:** Philip Salu, Katie M. Reindl

**Affiliations:** Department of Biological Sciences, North Dakota State University, Fargo, ND USA

**Keywords:** Anterior gradient 2 (Agr2), Protein disulfide isomerases, Biomarker, Cancers, Endoplasmic reticulum stress, Protein synthesis

## Abstract

Protein folding is an essential component of protein biosynthesis, allowing for post-translational modifications that ensure proper protein structure and function to support cellular physiology. The presence of unfolded proteins triggers cellular mechanisms to either remove the unfolded proteins or reduce protein synthesis. However, the accumulation of improperly folded proteins may lead to diseases, including neurological disorders and cancers. Indeed, cancer cells have a dysregulated protein synthesis capacity that enables them to survive in higher proliferative and growth states. The anterior gradient 2 (Agr2) protein is often overexpressed in multiple cancers to support the need for increased protein synthesis resulting from uncontrolled cell proliferation. Agr2 acts like a protein disulfide isomerase (PDI), catalyzing the formation of disulfide bonds in native proteins. Its expression in cancers has been associated with increased cell proliferation, metastasis, and invasion. Conversely, the lack of Agr2 has been associated with ER stress (ERS) and the activation of the unfolded protein response (UPR) pathway to restore cellular protein homeostasis. Furthermore, Agr2 can be secreted into the extracellular environment and has been detected in human urine and serum, highlighting its potential use as a cancer biomarker. This review discusses Agr2 and its role in protein synthesis and ERS. We examine recent developments regarding its detection and use as a biomarker and delve into emerging therapeutic strategies focused on targeting Agr2.

## Introduction

Protein synthesis is a tightly regulated process that plays a crucial role in preventing the development of neurodegenerative disorders such as Huntington’s disease (HD), Alzheimer’s disease (AD), amyotrophic lateral sclerosis (ALS), and various types of cancers [1–3]. However, protein synthesis is aberrantly upregulated in cancer cells due to high energy demands and the need to support increased cell growth and proliferative capacity [2, 4, 5]. As protein synthesis imposes a high energy demand on biosynthetically active cancer cells, they hijack and deregulate translational control mechanisms to improve their oncogenic impacts [6, 7]. With increasing protein demands, especially by cancer cells, comes the increased necessity for proper protein folding to ensure that the proteins maintain their correct conformation and functional integrity [8]. Protein folding, particularly oxidative protein folding, involves disulfide bond formation in native proteins [9, 10]. In eukaryotes, approximately 30% of all proteins have one disulfide (S–S) bond [11]. Disulfide bond formation may occur via either thiol oxidation or disulfide isomerization of non-native disulfide bonds formed in the early protein folding process [12, 13]. Disulfide bonds are critical for a protein’s stability, and the process is facilitated by oxidases and isomerases through thiol-disulfide exchange reactions, as shown in the reaction equation below [10, 14, 15]:

$${\text{P}} - \left( {{\text{SH}}} \right)_{2} + {\text{E}} - {\text{S}}_{2} \rightleftharpoons {\text{P}} - {\text{S}}_{2} + {\text{E}} - \left( {{\text{SH}}} \right)_{2}$$

P − (SH)_2_: Protein substrate with a dithiol bond; E − S_2_: Enzyme responsible for catalyzing disulfide exchange reaction (oxidized); P − S_2_: Protein product with disulfide bond; E − (SH)_2_: Disulfide bond transfer enzyme (reduced).

Anterior gradient 2 (Agr2), also known as PDIA17, belongs to the protein disulfide isomerase (PDI) family of proteins [16]. Its primary functions involve catalyzing the formation of disulfide bonds and acting as a chaperone to ensure proper folding of nascent proteins and assisting with protein quality control in the endoplasmic reticulum (ER) [17]. Agr2 is overexpressed in multiple cancers and is thought to help cells maintain protein homeostasis [18–21]. In addition to its role in protein folding, Agr2 is known to be involved in other cellular signaling pathways [22]. Its overexpression has been associated with tumorigenesis, cancer progression, and resistance to chemotherapy [22, 23]. The Agr2 protein can exist in a dimeric, ER-resident form or in a monomeric, secreted form [24, 25]. Secreted Agr2 has been detected in the extracellular environment of cells and in human serum and urine [26–28]. The unique existence of Agr2 in two different forms highlights its importance in both normal and disease tissues. Despite this, further research is necessary to better understand its role in disease and develop new biomarkers that can predict prognosis and response to chemotherapy. This review discusses the structure of Agr2, highlighting the significance of the two forms of Agr2, as well as other genetic or structural variants. We also examine recent developments regarding the involvement of Agr2 in disease development, progression, cellular signaling, and proteostasis. Finally, we review Agr2’s potential as a biomarker in disease detection and monitoring, current inhibitors, and their therapeutic implications.

## Genetic overview and protein structure of human Agr2

**Fig. 1:**
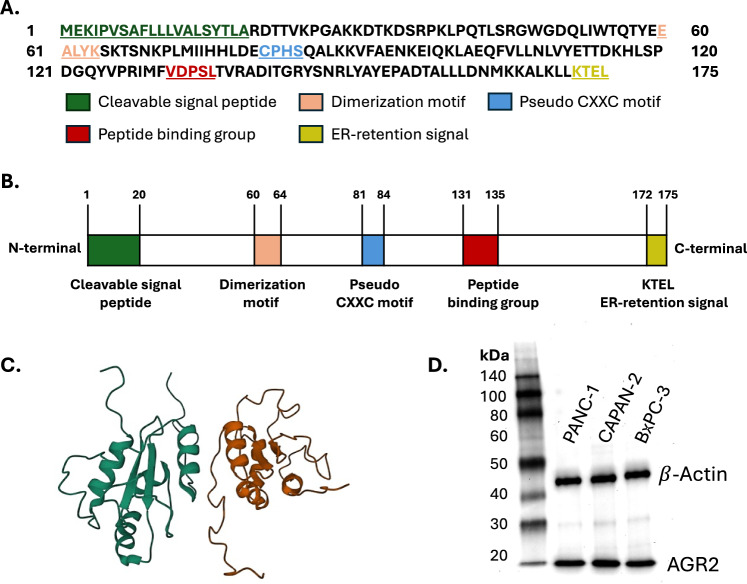
Structure of Agr2. **A** Amino acid sequence of the Agr2 gene highlights the presence of different motifs. **B** A full-length depiction of the structure of the Agr2 protein shows different motifs and terminals. **C** Protein structure of an Agr2 dimer obtained from the Protein Data Bank (PDB) database. **D** Western blotting shows the molecular weight of the Agr2 protein in kDa

The Agr2 gene is located at position 21 on chromosome 7 in humans [29]. It spans a region of 50 kb in genomic DNA and contains 7 transcripts with 210 orthologues and 2 paralogues [30]. The gene is also known as AG2, hAG-2, Xenopus anterior gradient 2 (XAG-2), and PDIA17 [30]. It encodes a protein that belongs to the PDI family and is predominantly localized in the ER [16]. The protein structure comprises approximately 175 amino acids (AA) (Fig. 1A). It has an ER signal peptide at the N-terminus, a dimerization motif, a catalytically active thioredoxin-like domain (pseudo-CXXC motif), a peptide binding group, and a C-terminal ER retention signal (Fig. 1B). The signaling peptide at the N-terminus helps in targeting the protein to the ER through a mechanism involving signal peptide-directed translocation of newly translated proteins [31]. This signaling peptide is also cleavable, allowing for Agr2 to be secreted out of the ER [28]. The presence of a dimerization motif enables two Agr2 protein monomers to interact, forming a dimer (Fig. 1C). This was confirmed using nuclear magnetic resonance to resolve the structure of Agr2, where the results showed that Agr2 predominantly exists as a homodimer [32]. The thioredoxin-like domain in Agr2 is characterized by a cysteine and a serine residue separated by two other amino acid residues instead of the typical cysteine-XX-cysteine residues [33–35]. The motif is responsible for catalyzing disulfide bond formation and isomerization, which is typical of proteins involved in redox regulation and protein folding [9, 36]. In addition, Agr2 also has the unique role of acting as a molecular chaperone [37]. It contains the amino acids VDPSL at positions 131 to 135 within its structural loop, which allows it to bind other peptide motifs (Fig. 1A). The chaperone function of Agr2 assists in the correct folding of proteins and the maintenance of cellular homeostasis [38]. At its C-terminal exists a transmembrane domain (KTEL) for membrane anchoring and retention in the ER [39]. This ER localization signal is required for Agr2 to be functionally active in the ER [39, 40]. Deleting the KTEL motif results in Agr2 secretion and loss of Agr2 function in the ER [41]. The human Agr2 protein is relatively small, with an observed molecular weight between 17 and 20 kDa (Fig. 1D). The data was generated using protein extracts from PANC-1, CAPAN-2, and BxPC3. The Agr2 primary antibody, Cat. no. 13062 was obtained from Cell Signaling Technologies.

Agr2 belongs to the anterior gradient (Agr) family of proteins. These proteins are also protein disulfide isomerases (PDIs) with several structural and functional similarities, including size and molecular mass [42]. However, Agr2 has a unique primary protein structure, ER retention signal, secretory signal, and PDI active site. In the next section, we discuss these similarities and differences that make Agr2 unique compared to other protein family members.

## Genetic and structural variants of AGR2

Anterior gradient proteins (AGRs) are a family of proteins involved in various cellular processes, including protein folding through disulfide bond isomerization in the ER [43, 44]. Currently, there are three known proteins in the AGR family: Agr1, Agr2, and Agr3 [42]. These proteins share certain sequence-specific homologies and are considered paralogues (Table 1). Aligning the amino acid sequences of these paralogues shows similarities in their catalytically active thioredoxin-like motif and the ER retention signals. The Agr1 gene has a classic thioredoxin fold motif (CXXC motif) and its protein has the ability to form mixed disulfide bonds in the ER [45, 46]. On the other hand, the consensus sequences of the thioredoxin motif in Agr2 and Agr3 lack one cysteine in their active sites [32, 47]. The cysteine is instead replaced by serine, a change that might affect the thiol-reactive oxidative functions of these proteins [32, 47]. Nonetheless, Agr1, Agr2, and Agr3 all have ER retention signals EDEL, KTEL, and QSEL, respectively, allowing for their retention in the ER (Table 1).

**Table 1:** Sequence similarities of Agr1, Agr2, and Agr3

	AA length	Cleavable signal	Dimerization motif	Thioredoxin-like motif	Peptide binding	ER retention signal
Agr1	172	METRPRLGATCLLGFSLLL	DGKKE	CGHC	LDPSG	EDEL
Agr2	175	MEKIPVSAFLLLVALSYTLA	EALYK	CPHS	VDPSL	KTEL
Agr3	166	MMLHSALGLCLLLVTVSSNLA	EGLFY	CQYS	VDPSL	QSEL

Amino acid (AA) sequences were obtained from NCBI, and alignments were done using the blastp suite on the NCBI blast platform. FASTA sequences of Agr1 and Agr3 were aligned against Agr2

**Fig. 2:**
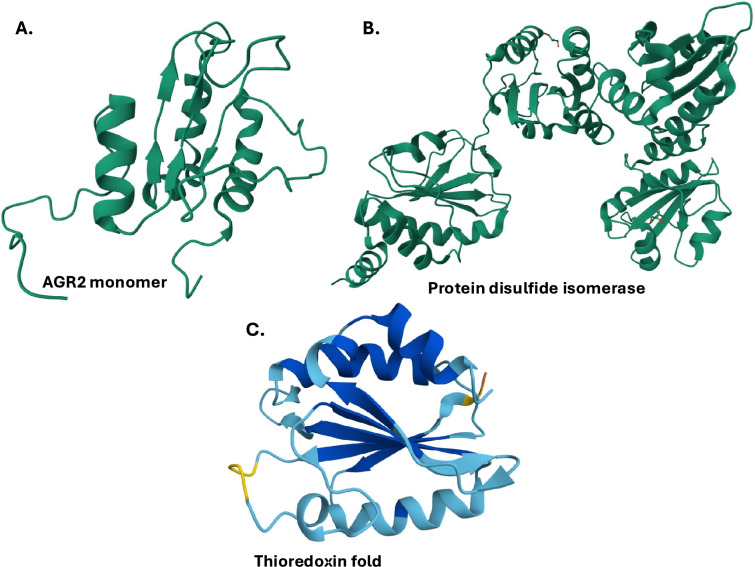
Structures of AGRs, PDIs, and the thioredoxin superfamily of proteins. **A** Agr2 monomer showing the presence of a thioredoxin-like fold. **B** PDI showing four thioredoxin folds. **C** Oxidized thioredoxin consisting of four $\beta$-sheets surrounded by three $\alpha$-helices. Images were created using the AlphaFold database [53]

AGRs are proteins that share similarities in function and sequence to other proteins groups. Functionally, AGRs belong to the PDI protein family. PDIs have catalytic activities ranging from thiol-disulfide oxidoreductase to disulfide isomerization and redox-dependent chaperone functions [16, 17]. The AGRs also function as chaperones due to their peptide-binding activity mediated by their peptide-binding groups [48]. All members of the PDI family share a thioredoxin-like domain structure of $\beta \alpha \beta \alpha \beta \alpha \beta \beta \alpha$ folds [49, 50]. They mostly contain four thioredoxin-like domains in an *abb’a’* arrangement [51]. The a and a’ domains contain the two redox-active sites (CXXC motifs) linked by the inactive b and b’ domains [51]. The presence of thioredoxin motifs in AGRs and PDIs means that these proteins are also considered to be part of the thioredoxin superfamily (TSF) of redox proteins [51]. Figure 2 below shows some similarities between AGRs, PDIs, and the thioredoxin superfamily of proteins. Table 2 summarizes the genetic and structural variants of Agr2, PDIs, and the TSF in terms of their AA size, active-site sequence, TRX-like domain, and ER retention sequences [16, 17, 52].

**Table 2:**
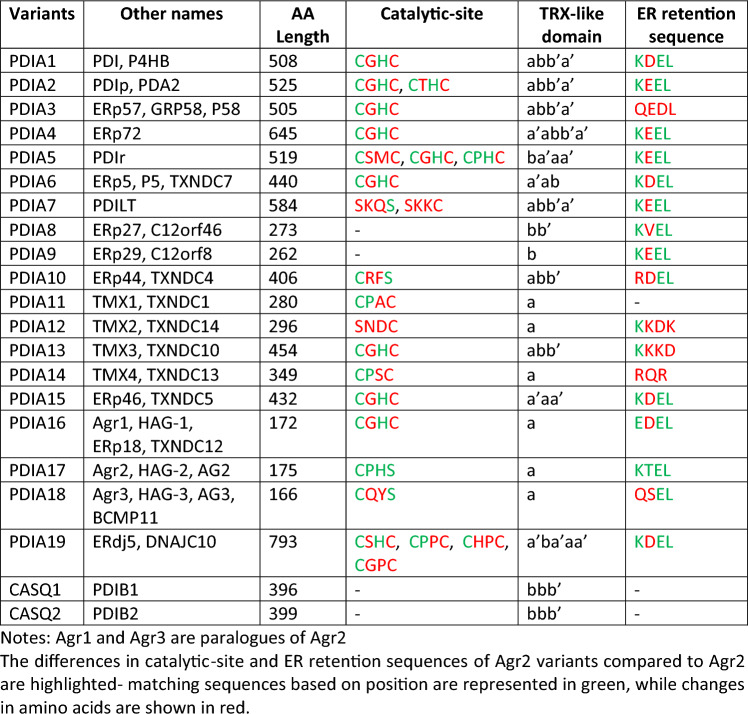
Structural variants of the Agr2 gene and their functions

## Prognostic impact of Agr2 expression in cancer

**Fig. 3:**
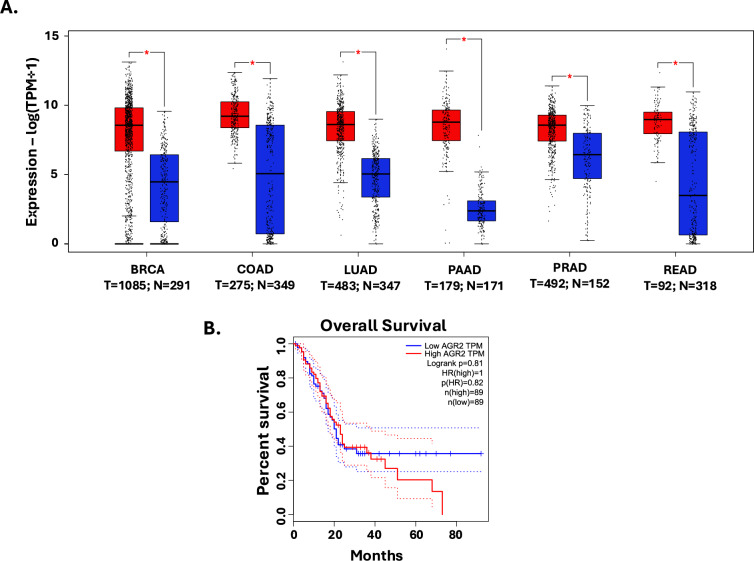
Agr2 is over-expressed in different cancer types. **A** Agr2 is differentially upregulated in different cancers. The number of tumor samples (T) and normal samples (N) used for each analysis are indicated. **B** Increased Agr2 expression is associated with decreased patient survival. Agr2 expression was determined from TCGA PAAD data compared to matched normal samples using GEPIA [55]

The expression of Agr2 in cancer was first detected in estrogen receptor-positive (ER +) breast cancer cells [54]. Since then, the expression of Agr2 has been shown to be elevated in various adenocarcinomas, including breast (BRCA), colon (COAD), lung (LUAD), pancreas (PAAD), prostate (PRAD), and rectum adenocarcinoma (READ) (Fig. 3A). Its overexpression correlates with decreased patient survival (Fig. 3B).

Even though Agr2 is expressed in normal cells or tissues, increased expression has been associated with the initiation and development of cancer [56]. This abnormal expression significantly alters cell fate by promoting cancer cell survival [22]. In gastric cancer, overexpression of Agr2 was associated with rapid disease progression and poor patient survival [57]. Agr2 also plays a critical role in cellular processes such as cell proliferation, migration, and metastasis [58–60]. Although its involvement in these processes is not fully understood, increasing research continues to delve into the mechanistic underpinnings of Agr2 and its potential roles in mediating different cell fate decisions. In LUAD cells, increased expression of Agr2 was associated with hypomethylation of CpG islands and facilitated cell proliferation, migration, and invasion [61]. A genetic screen of metastatic lesions compared to matched primary tumor samples revealed an upregulation of Agr2 through epigenetic modification of its promoter region by a complex of the long non-coding RNA LINCO2273 and hnRNPL protein [62]. Also, lung microarrays showed Agr2 expression level was a significant predictor of overall survival in patients below 65 years [18]. Further, an analysis of primary prostate cancer showed significant Agr2 expression in about 93.5% of tissues. In colorectal cancer (CRC), tumor-associated neutrophils (TANs) secreted Agr2 in the tumor microenvironment, which promoted the migration of CRC cells [63]. Consequently, neutrophil-specific ablation of Agr2 decreased CRC liver metastasis in mice [63]. Contrastingly, Chevet and colleagues showed that Agr2 is exclusively expressed in tumor cells using immunohistochemistry staining compiled on more than 21 CRC samples, thus suggesting a direct role in tumor biology [64]. The studies also differ in their prognostic implications. While Tian et al. associated high Agr2 + TANs with poor prognosis, Chevet et al. linked high epithelial Agr2 expression with better patient outcomes. It is important, however, to recognize that Tian et al. focused on the extracellular signaling axis involving TANs and CRC cells, whereas Chevet emphasized the intracellular role of Agr2 within epithelial cells. Regardless, these discrepancies highlight the complexity of Agr2’s role in cancer and warrant the need for further research. In Moffitt’s classification of pancreatic ductal adenocarcinoma (PDAC) into classical and basal-like subtypes, Agr2 was one of the genes used to delineate a tumor type as a classical subtype [65]. This tumor subtype was characterized by an activated stroma, with patients having a worse median survival compared to patients with normal stroma [65]. In a recent preclinical study correlating Agr2 expression with clinicopathological data in a prostate cancer cohort, Agr2 expression was inversely related to patient survival. However, Agr2 was not suitable as an independent prognostic marker for prostate cancer patients [66].

## Agr2 and drug resistance

Overexpression of Agr2 has been shown to correlate with drug resistance in EGFR-mutant non-small cell lung cancer (NSCLC) [67] and postmenopausal breast cancer patients [68]. The roles of Agr2 in chemoresistance can vary depending on the type of cancer, but some potential mechanisms include promoting cancer cell survival, inhibiting apoptosis, enhancing DNA repair, and modulating signaling pathways involved in chemoresistance [69–71]. Agr2 might also be involved in cancer relapse or tumor escape [72]. Research has suggested that cancer cells can enter into a state of senescence, characterized by an irreversible cell cycle arrest and a cessation of cell proliferation due to cellular stress, DNA damage, telomere dysfunction, and chemotherapy [73, 74]. However, the senescent state is also associated with other cellular states, such as terminal differentiation, quiescence, dormancy, or drug-tolerance persistence [75–77]. Although chemotherapy can induce cellular senescence, the presence of senescent cells that survive successive chemotherapy regimens can create survival niches through paracrine cooperation with adjacent cells, thereby enabling chemotherapy escape. In this regard, Agr2 has been suggested to induce the proliferation of senescent cells via the activation of the mammalian target of rapamycin (mTOR)/AKT signaling pathway [72]. This has the potential to cause relapse and increase chemotherapy resistance. On the other hand, Agr2 knockdown using short interfering RNA (siRNA) was shown to reduce chemotherapy resistance and induce cell apoptosis with the involvement of the ERK/AKT pathway [69]. Short hairpin RNA (shRNA) mediated knockdown of Agr2 in the ampulla of Vater cancer cell lines also led to decreased cell viability and anchorage-independent growth by up to 98% [78]. Interestingly, the knockdown cells failed to form detectable tumor xenografts in nude mice [78]. Moreover, gene knockdown increased the sensitivity of cells to gemcitabine, 5-fluorouracil (5-FU), and cisplatin [78]. On the contrary, Agr2 expression has been shown to promote cisplatin resistance by activating the AKT signaling pathway and enhancing SREBP2-mediated cholesterol metabolism [79]. Other studies have also used Agr2 knockdown models to therapeutically profile cell response to enhance the clinical utility of Agr2 knockdown [69, 78, 80]. For instance, Agr2 silencing contributed to metformin-dependent sensitization of colorectal cancer cells to 5-FU and oxaliplatin treatment [80]. Overall, Agr2 is overexpressed in most cancers and affects cell proliferation, migration, metastasis, and drug resistance. Its involvement in multiple pathways, including those that regulate cell growth, differentiation, and apoptosis, as well as interactions with other proteins, is being investigated. This offers a promising avenue for Agr2 targeting as a potential cancer treatment option.

## Molecular mechanisms of Agr2 interactions

Agr2 is known to interact with several proteins, and these interactions have been found to play important roles in various cellular processes including protein folding, secretion, and cell signaling [81]. Agr2 is an ER-localized molecular chaperone that is involved in modulating ER stress, the UPR pathway, chemoresistance, and cell signaling [22, 40, 82]. Agr2 interacts with different signaling molecules and pathways, such as the ERK, PI3K/Akt, and Wnt pathways, and influences cell proliferation, survival, and migration in cancer [69, 83, 84]. Indeed, emerging research has revealed that Agr2 knockdown significantly decreases cell viability, migration, and invasion [85, 86]. Following Agr2 knockdown, Jin and colleagues observed decreased N-cadherin and increased expression of E-cadherin, α-sma, and vimentin [85]. Furthermore, deletion of Agr2 inactivated the TGF-beta/SMAD signaling pathway, thereby highlighting Agr2 as a promoter of epithelial-mesenchymal transition (EMT) through the activation of TGF-beta/SMAD signaling [85]. Our own analysis of Agr2 knockdown in pancreatic cancer cells revealed an increase in mitochondria fission via increased expression of mitochondrial fission factor (MFF) and phosphorylation of dynamin-related protein 1 (pDRP1) [86]. Agr2 has also been implicated in the activation of cancer-associated fibroblasts (CAFs) through Wnt signaling [87]. The activated CAFs then secrete insulin-like growth factor 1 (IGF1), which enhances Agr2 expression and secretion through the IGF1-receptor/c-Jun axis, thereby intensifying desmoplasia, immunosuppression, and tumorigenesis [87]. Also, Agr2 positively regulates programmed cell death ligand 1 (PDL1) in colorectal cancer cells (SW480 and SW620), effectively paving the way for immune evasion of the cells [88].

**Fig. 4:**
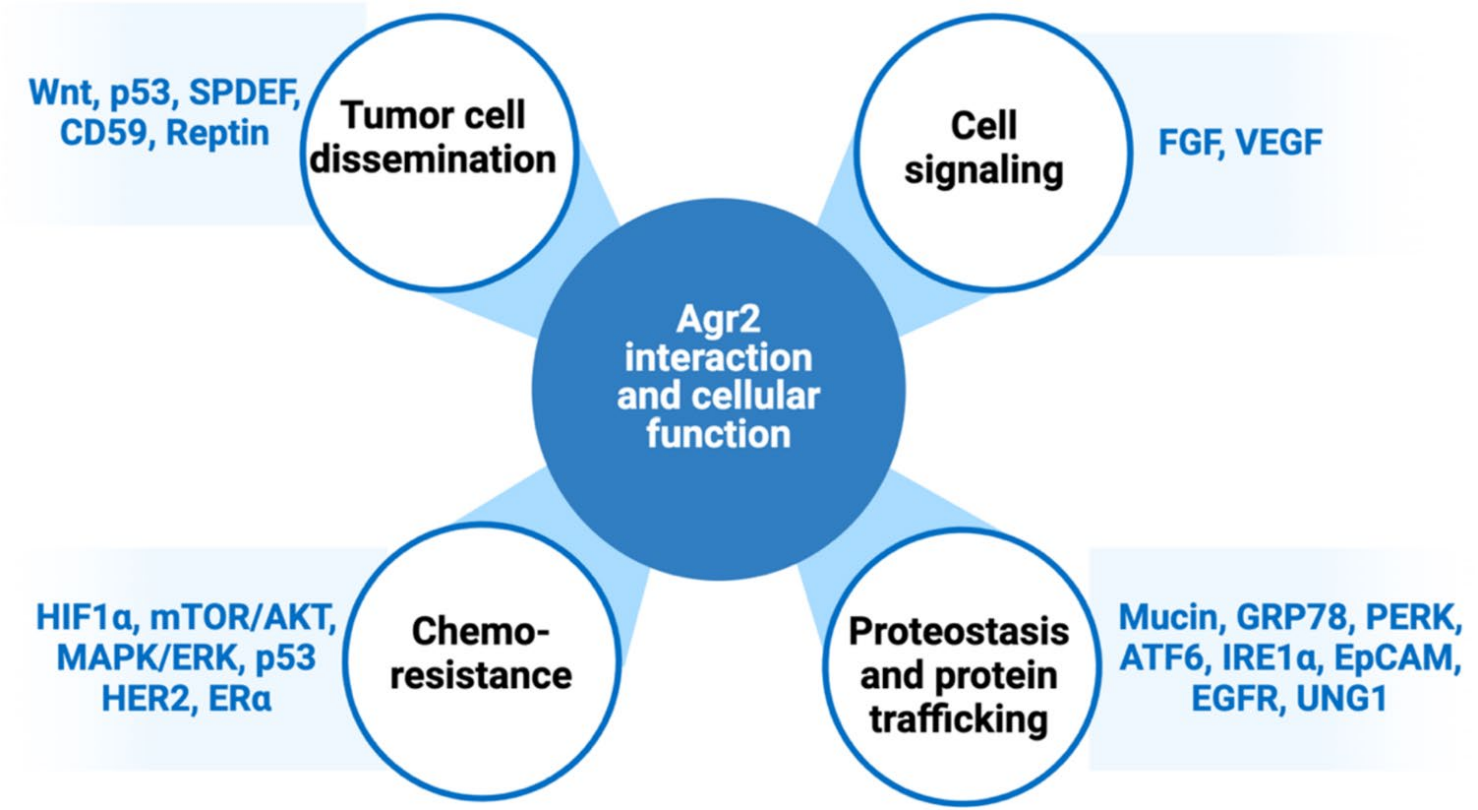
Effects of Agr2 on cellular function, protein targets, and interaction partners. Agr2 is involved in tumor initiation and dissemination, cellular signaling to mediate different cell-fate decisions, regulating proteostasis and protein trafficking, and chemoresistance

Additionally, Agr2 is involved in protein trafficking and the assembly of cysteine-rich transmembrane receptors and the cysteine-rich intestinal glycoprotein mucin [89–91]. Figure 4 shows the effects of AGR2 on cellular function and related protein targets. Previous reviews have characterized these AGR2 interactions more thoroughly [24, 38, 90]. Although further research is needed to define the Agr2 interactome, understanding its functions and potential implications in various diseases could provide valuable insights.

### Agr2, ERS, and the UPR pathway

Protein folding is necessary in eukaryotes to allow for proper protein conformation and function [10]. However, protein folding can be affected by cellular environmental factors such as pH, temperature, stress, and gene mutations [92, 93]. The presence of misfolded protein aggregates often serves as a conduit to many diseases, including cancers [94]. As a PDI primarily localized to the ER, Agr2 plays a role in protein folding, and several studies have suggested a connection between Agr2, ER stress, and the UPR pathway [21, 52, 95]. Indeed, dysregulation of Agr2 expression or function has been linked to ERS and UPR activation [95]. UPR works by either lowering the number of proteins that need folding, increasing the folding capacity of the ER, or removing unfolded proteins [96]. Misfolded proteins are often cleared or destroyed through the ubiquitin-proteosome system [97, 98]. However, continuous accumulation of unfolded or misfolded proteins leads to stress referred to as proteotoxic stress [99]. Sustained proteotoxic stress leads to the dysregulation of signaling pathways controlling cell survival and death [100]. Research indicates that Agr2 may modulate the UPR pathway by influencing the activation of key UPR sensors like double-stranded RNA-activated protein kinase R-like ER kinase (PERK), inositol-requiring enzyme 1 (IRE1) and activating transcription factor 6 (ATF6) [101, 102].

### Agr2 and p53

Tumor protein 53 (TP53), also known as p53, is a tumor suppressor protein that regulates cell division and prevents cells from proliferating uncontrollably [103]. Mutated p53, on the other hand, acts as a dominant inhibitor of wild-type p53, promoting tumor growth [104]. Loss of p53 function is often considered a prerequisite for cancer development [103]. Approximately 50% of human cancers carry a loss-of-function mutation in the p53 gene [105]. Although the p53 mutational spectrum differs among tumors, mutations in p53 are linked to poor prognosis in malignancies of breast, bladder, and pancreas [106–109].

There is evidence that Agr2 inhibits p53 activity and loss of Agr2 expression activates p53 signaling. In TP53 wild-type esophageal squamous cell carcinoma (ESCC), the down-regulation of Agr2 led to reduced cell proliferation and an increased phosphorylation of p53. This effect was not observed in mutant p53 ESCC cells, indicating that Agr2 could enhance cell growth by inhibiting p53 phosphorylation in TP53 wild-type ESCC, but not in TP53 mutant ESCC [110]. An investigation by Hrstka et al. showed that Agr2 influences p53 activation in cancer cells [111]. They showed that increased Agr2 leads to elevated DUSP10, a phosphatase that deactivates p38 MAPK, a well-known regulator of p53 through post-translational modifications that enable stabilization and activation of p53 [111]. In glioblastoma cells isolated from mouse and human tumors, ER protein reflux to the cytosol occurs upon ER proteostasis perturbation. Under ER stress, Agr2 is released from the ER and localizes to the cytosol, where it gains new functions by interacting and inhibiting wild-type p53 activity, thus providing a selective advantage for tumor cells [112]. A pre-clinical study that targeted Agr2 expression using the monoclonal antibody mAb18A4 demonstrated an activation of the p53 pathway, further highlighting a possible interaction between Agr2 and p53 [113]. Treatment with the antibody activated p53 pathway and attenuated ERK1/2 MAPK pathway leading to enhanced apoptosis, attenuated proliferation, reduced tumor size and increased survival of xenograft tumor models [113].

### Agr2 and mucins

Agr2 is a protein that plays a regulatory role in the production of mucins, which are glycoproteins responsible for forming a protective barrier in the body, particularly in the mucous membranes lining various organs like the respiratory and digestive tracts [114, 115]. In some cancers, Agr2 has been found to influence the production and secretion of mucins, potentially impacting the development and progression of disease [116]. A study using Agr2 null mice revealed a poorly developed inner colon mucus layer filled with fewer goblet cells [95]. In 2009, Park and colleagues observed the importance of Agr2 in intestinal mucus production [89]. In particular, the concentration of secreted Agr2 was high in intestinal mucus [117]. Intriguingly, Agr2 was also identified as a mediator of MUC2 processing and secretion [116, 117]. Recently, Wu et al. discovered the mechanism of MUC2 processing by Agr2, whereby glutamine promotes O-linked N-acetylglucosamine (O-GlcNac) modification of glucose-6-phosphate dehydrogenase (G6PD) through the hexosamine pathway, thereby promoting MUC2 maturation [118]. Furthermore, Agr2 could bind to MUC1 and induce the expression of hypoxia-inducible factor 1$\alpha$ (HIF-1$\alpha$), leading to the regulation of glucose metabolism [119]. Tonelli and colleagues discovered Agr2 to have a tumor-promoting function in a recent study describing a mucus production program that facilitates subtype interconversion from classical towards basal-like differentiation in pancreatic ductal adenocarcinoma [120]. They showed that Agr2 was regulated by the transcription factor SPDEF which is highly active in precancerous lesions and maintains the classical subtype of PDAC. Importantly, the SPDEF tumor-promoting function is impaired when Agr2 is deleted, consequently reducing tumor growth in vivo [120]. In an in vivo study using a CRC model, Agr2 was shown to promote metastasis and negatively regulate activity of the canonical β-catenin pathway by activating CAMKII-dependent non-canonical Wnt-signaling [121].

## Agr2 as a predictive biomarker

Biomarkers are becoming increasingly popular as tools for predicting disease prognosis and response to therapy in patients. Considerably, the focus of ongoing research is detecting and identifying noninvasive biomarkers in patients. Agr2 is mainly localized in the ER; however, its structure shows that it can be secreted by cells, particularly cancer cells. Cell-based analysis of Agr2 has revealed its presence in different subcellular locations, such as the cytoplasm and plasma membrane [40]. In cell culture, Agr2 was detected in both cells and their growth media, further indicating that cells can secrete Agr2 [122].

Consequently, Agr2 is not only present in tumor tissues but is also secreted in bodily fluids like blood, urine, and saliva and may serve as a convenient and accessible biomarker for cancer diagnosis [123–125]. The mechanisms underlying Agr2 secretion into bodily fluids remain a topic under investigation. However, emerging evidence suggests that the secreted form of Agr2 is mostly its monomeric form, with the dimers mostly localized to the ER [25]. Also, a loss of the ER retention motif may lead to the secretion of Agr2 [41].

Indeed, serum Agr2 has been shown to be a useful biomarker in pituitary adenomas (PAs) [125]. A study conducted on 163 PA patients, 7 prostate cancer patients, 43 patients with other sellar lesions excluding PAs, and 20 normal individuals showed significantly higher levels of serum Agr2 in PA patients (250.10 ± 79.14 ng/ml) than in patients with other sellar lesions (220.84 ± 79.62 ng/ml, *P* = 0.017) and normal individuals (163.67 ± 50.38 ng/ml, *P* < 0.001) [125]. Additionally, serum Agr2 holds diagnostic value in nasopharyngeal carcinoma prognosis and epithelial and mucinous ovarian cancers [42, 126, 127]. Moreover, elevated Agr2 levels are linked to resistance to certain cancer-targeted chemotherapies. In ER-positive breast cancer patients, serum Agr2 was used as a positive predictor for tamoxifen resistance [128]. Likewise, in EGFR-mutant NSCLC cells, increased Agr2 was associated with resistance to gefitinib [67]. This suggests that AGR2 could be a biomarker to predict responses to specific treatments and guide personalized therapy decisions. Unsurprisingly, Agr2 secretion has been demonstrated to promote tumorigenic properties by regulating the microenvironment of epithelial tissue and facilitating the acquisition of invasive and metastatic characteristics [122].

Nonetheless, current limitations involve issues with the specificity and sensitivity of Agr2 detection methods. As shown in Fig. 3, Agr2 is overexpressed in different cancer types, making it challenging to use as a definite biomarker for a single disease. To date, no detection method has been developed that is sensitive enough to measure Agr2 in human secretions accurately, thereby limiting its clinical utility. A study using RT-PCR and ELISA was able to measure Agr2 mRNA levels in tissues and in the serum of healthy donors, breast cancer and nasopharyngeal cancer patients [127]. Even though the assays detected significant differences (*P* < 0.05) in Agr2 levels between healthy and diseased individuals, the detected levels failed to provide any clinical advantage for differential disease diagnosis [127]. In this regard, future research should be directed at improving the sensitivity and specificity of Agr2 detection methods. Efforts aimed at using biosensors have enabled the detection of femtogram levels of Agr2 using monoclonal antibody modified screen-printed gold electrodes [129]. Some research has also focused on combining Agr2 with other biomarkers using multi-panel assays [130–132]. In estrogen receptor-positive breast cancer, Agr2 in combination with FOXA1 was a positive prognostic indicator of disease, and low levels of both Agr2 and FOXA1 were associated with best prognosis compared to any other combinations [130]. Similarly, in distinguishing lung squamous cell carcinoma (SCC) from adenocarcinoma (ADC), a combination of Agr2 and KRT5 in a validation dataset had a 100% predictive accuracy for ADC compared to an 86.7% accuracy for SCC [131]. These combinations can provide better specificity and improve the accuracy of disease detection, as well as help in distinguishing between different types of cancers.

With advancements in assay sensitivities, efforts targeted at evaluating Agr2 concentrations in human serum and other bodily fluids hold promise to define Agr2’s utility in disease diagnosis and monitoring. Further studies are needed to fully understand the mechanistic underpinnings for Agr2’s presence in different subcellular compartments and its secretion. In the future, assay validation, improved sensitivity, and specificity tests will be needed to detect Agr2 and increase its effectiveness as a biomarker for disease diagnosis and monitoring.

## Inhibitors of Agr2 and therapeutic implications

**Fig. 5:**
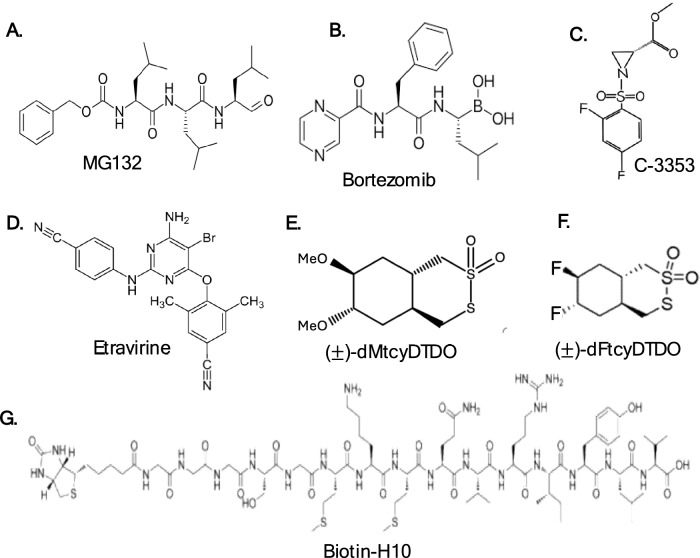
Structure of chemicals or molecules that bind to and inhibit the activity of Agr2. **A** MG132 is a tri-peptide aldehyde with a membrane-permeable proteasome inhibitory function. **B** Bortezomib is a first-in-class ubiquitin–proteasome inhibitor used to treat multiple myeloma and mantle cell lymphoma. **C** C-3353 is an aromatic N-sulphonamide derivative of aziridine-2-carboxylic acid with inhibitory effects against different PDI isoforms. **D** Etravirine is a non-nucleoside reverse transcriptase inhibitor that indirectly inhibits Agr2. **E** and **F** Disulfide bond disrupting agents that bind to the active site of Agr2 **G** Biotin-H10 is a biotinylated peptide that binds and directly inhibits the activity of Agr2

The importance of Agr2 in different cellular functions, along with the correlation of its elevated levels with different pro-oncogenic features, highlights its relevance as an anti-tumor target. Although the Agr2 gene has a druggable structure, there are no FDA-approved drugs or bioactive compounds that specifically target it. However, there has been a growing interest in regulating Agr2 activity through small molecule inhibitors, clinical drugs, biologics, and shRNA-mediated mechanisms [69, 133, 134]. A commonly used inhibitor of Agr2 is the cell-permeable proteasome inhibitor MG132 (Fig. 5A), a peptide aldehyde that facilitates Agr2 degradation through polyubiquitination and the activation of autophagy [135]. It was found to cause a remarkable decrease in the expression of Agr2 at both the mRNA and protein levels in the NSCLC cell line, A549 cells [135]. Another proteasome inhibitor, bortezomib (Fig. 5B), exerted similar inhibitory effects on the expression of Agr2 [135]. Bortezomib is a first-in-class reversible and selective ubiquitin–proteasome pathway inhibitor that degrades many intracellular proteins. It is commonly used in the treatment of both refractory or relapsed multiple myeloma and, more recently, mantle cell lymphoma. Importantly, Agr2 inhibition using either MG132 or bortezomib significantly enhanced the anti-tumor efficacy of bevacizumab, indicating the importance of Agr2 as a predictive marker for patient selection or stratification for chemotherapy [135].

### Small molecule inhibitors

Small molecule inhibitors are being explored to directly inhibit Agr2 or pathways associated with Agr2 function. Recently, aromatic N-sulphonamides of aziridine-2-carboxylic acid-derivates were developed and have shown inhibitory properties against different PDI isoforms [136]. In particular, C3353 (Fig. 5C) was shown to degrade the Agr2 protein, leading to the inhibition of cell adhesion to collagen at a half-maximal inhibitory concentration (IC50) of 20.1 µM in a hormone-sensitive, high Agr2-expressing breast cancer cell line MCF-7 but not in the triple-negative, low Agr2 expressing cell line MDA-MB-231 [137]. This result suggests that targeting Agr2 might have anti-cancer effects, particularly in highly malignant cancer types, by regulating cancer cell adhesion. Another inhibitor of Agr2 is Etravirine (Fig. 5D), a non-nucleoside reverse transcriptase inhibitor [138]. This compound was found to induce Agr2 degradation through autophagy, leading to decreased cell proliferation, migration, and invasion of ovarian cancer cells in vitro [138]. A combination of etravirine and paclitaxel showed significant suppression of tumor progression and metastasis in vivo in an orthotopic xenograft mouse model [138]. Furthermore, disulfide bond-disrupting agents (DDAs) have also been observed as active site inhibitors of Agr2. Using biotinylated-DDA-based affinity pulldown techniques, Law and colleagues detected monomeric Agr2 as a binding partner of DDAs [133]. Examples of such DDAs are dMtcyDTDO and dFtcyDTDO (Fig. 5E and F), which inhibited Agr2 and induced apoptotic cell death associated with ERS, disulfide bond mediated oligomerization of Death Receptors 4 and 5 (DR4/5), and EGFR, and upregulation of DR5 [133, 139, 140]. Biotin-H10 (Fig. 5G) is another inhibitor of Agr2 that has been shown to decrease the viability of cancer cells [141]. It has a high affinity for Agr2 with a dissociation constant (K_D_) of 7.5nM [141]. This peptide inhibitor selectively binds and interacts with two regions of Agr2, namely the amino acid residues surrounding 47–60 and 97–116 [141]. Compared to the monomers, Agr2 dimers exhibited high affinities to Biotin-H10, which could potentially impact the inhibition of secreted Agr2 [141].

### Antibody/ RNA interference inhibitors

Additionally, monoclonal antibodies are currently being developed to target Agr2 [142, 143]. For instance, the anti-Agr2 monoclonal antibody, mAb18A4, was found to inhibit lung cancer progression and metastasis in lung adenocarcinoma xenograft models and decreased proliferation and colony formation with increased apoptosis in cancer cells [113]. Mechanistically, treatment with this antibody activated the p53 pathway and attenuated the ERK1/2-MAPK pathway [113]. Targeting Agr2 with antibody-based therapies is of utmost importance in the development of effective treatments. By utilizing such therapies, it is possible to minimize the risk of off-target side effects, making it a highly promising avenue for research. Some studies have investigated the effects of inhibiting Agr2 using short hairpin RNA (shRNA) or small interfering RNA (siRNA) molecules [144, 145]. In a colorectal cancer cell model, silencing Agr2 contributed to metformin-dependent sensitization of cells to chemotherapy [80]. This implies that while suppressing Agr2 can impede the progression and spread of tumors, it could also make cancer cells more receptive to chemotherapy, thereby leading to better outcomes for patients.

It is worth noting that targeting Agr2 and Agr proteins can produce unintended biological effects due to their expression patterns and involvement in normal physiology. As a critical factor in mucin production and secretion in the gastrointestinal tract, disruption of Agr2 may impair mucus production, which may increase the risk of infection [120]. Given its role in maintaining ER homeostasis, abrupt disruptions in Agr2 protein expression may lead to ER stress, and even cell death in otherwise healthy cells. Small molecule inhibitors or antibodies designed to target Agr2 may also cross-react with other Agr family members, thereby affecting unrelated pathways. Indeed, Agr2 knockdown has been shown to lead to increased expression of compensatory ER-stress related genes such as PDI, IRE-1, and PERK [86].

## Future directions

Agr2 is involved in protein post-translational modification and the maintenance of protein homeostasis. It is particularly involved in the redox folding of proteins in the ER. The expression of Agr2 has been linked to various pro-oncogenic signatures, including cancer progression, metastasis, and drug resistance, and correlates with poor patient survival. Although Agr2 is overexpressed in multiple cancer types, its expression varies in different cancer cell lines and patient samples or tumors. This peculiar expression pattern forms a basis for using Agr2 as an anti-cancer target for selected cancers and patients. Besides, Agr2 can be secreted into patients’ serum. Thus, detecting Agr2 in patients with cancer can serve as a prognostic indicator of disease occurrence, progression, and response to chemotherapy. Regardless, more in-vivo research is needed to validate the mountain of in vitro data on the effects of Agr2 overexpression. This will also need to be followed with clinical validation test to further improve the diagnostic value of Agr2. Also, detection methods need to be developed and optimized to provide noninvasive and easily accessible screening options. Currently, no FDA-approved drugs target Agr2 despite evidence showing that the gene is druggable. Research is needed to design and test specific drugs targeting Agr2, which should have the potential to enter clinical trials. These drugs can also be used to sensitize tumors in order to allow for other standardized chemotherapy regimens.

## Conclusion

Recent evidence has highlighted the important roles of Agr2 in disease and cancer. Agr2 has been found to promote cell differentiation, proliferation, migration, and dissemination. It is also secreted into human urine and serum and has the potential to become an important cancer biomarker. Although there are currently no FDA-approved drugs specifically targeting Agr2, the outlook remains promising as increasing research is focused on determining modulators and Agr2-targeting drugs to help improve patient outcomes.

## Data Availability

No datasets were generated or analysed during the current study.
